# Healthcare professionals’ perceptions of patient safety in European emergency departments: a comparative analysis of survey results

**DOI:** 10.1007/s11739-023-03523-1

**Published:** 2024-01-26

**Authors:** Roberta Petrino, Carola Biondi, Luis Garcia Castrillo

**Affiliations:** 1 Emergency Medicine Unit, Department of Critical Care, Ente Ospedaliero Cantonale, Via Tesserete 46, 6900 Lugano, Switzerland; 2 Department of Emergency Medicine, Hospital Universitario Marques de Valdecilla, Santander, Spain

**Keywords:** Patient safety, Surveys and questionnaires, Emergency service, Hospital, Health personnel attitude

## Abstract

Patient safety is a significant concern worldwide. The Emergency Departments (EDs) are vulnerable to adverse events. Europe, with its diverse healthcare systems, differs in patient safety. This study aimed to identify safety challenges through a comparative analysis of healthcare professionals’ perceptions of patient safety in European EDs. In early 2023, a validated questionnaire was distributed to European ED professionals, meeting specific response rate criteria. The questionnaire included five safety domains and additional questions about infection control and team morale, with 36 ordinal scale questions. Responses ranged in five levels from “Never” to “Always,” and the scores were summed to calculate the total safety score (TSS). The study examined the impact of per capita healthcare expenditure on safety perceptions using descriptive statistics, correlation assessments and SPSS 17 used for the analysis. The analysis of 1048 valid responses from 24 European countries revealed significant variability in safety perceptions. Teamwork scored highest, signifying effective collaboration. Common safety issues included overcrowding, patient flow management, understaffing, limited training and facilities for mental illnesses. TSS showed correlation with team morale and infection control, but no correlation with per capita healthcare expenditure. This comparative study underlines the disparities in patient safety perceptions across European EDs. Each country displayed unique safety concerns. Safety perceptions did not align with per capita healthcare expenditure, indicating that addressing ED safety needs multifaceted strategies. Policymakers can leverage these findings to inform strategic planning, encouraging targeted interventions to enhance patient safety at both the national and European levels.

**Supplementary Information:**

The online version contains supplementary material available at 10.1007/s11739-023-03523-1.

## Introduction

The World Health Organization calls for patient safety to be recognized as a health priority in national health policies and programs [1]. Patient harm from unsafe care is a significant and growing public health concern globally, contributing substantially to mortality and disability rates worldwide [2–5], including Europe [6]. In high-income countries, clinical waste accounts for up to 15% of hospital spending due to safety failures [1]. The cost associated with medication errors has been estimated at $42 billion USD annually globally, excluding lost wages and productivity or increased healthcare costs [7]. The costs related to adverse events in European hospitals vary between 1.3 and 32% of public health expenditure [6].

Significant variations remain between healthcare systems in different European countries, in terms of both quality of care and patient safety [6, 8]. European countries adopt different ways of organizing and financing their healthcare systems; two common models are the public and private healthcare system. They also have different access to care, care processes, and healthcare spending per capita [9]. A considerable part of patient harm, and thus healthcare costs, can be avoided by promoting a culture of safety [6, 10]. Emergency departments (EDs) are at high risk of adverse events that affect patient safety [11–14]. Rapid patient turnover, overcrowding, and physician inexperience, which are common in EDs, can increase patient mortality [15, 16]. Furthermore, burnout in emergency medicine not only has serious consequences on the well-being of healthcare workers but can also negatively affect the quality and safety of care provided to patients [17, 18]. The effective management of medical emergencies requires robust and well-organized healthcare systems capable of providing timely, appropriate, and safe care to all patients. In early 2023, a validated questionnaire [19] was distributed to healthcare professionals working in EDs to assess their perceptions of safety. The global results have been published elsewhere [20]. This study aimed to conduct a comparative analysis of the results among ED workers in most European countries and highlight the most critical aspects related to safety in each country.

## Study design and methods

The researchers designed an observational study based on a cross-sectional online survey. Study participants were healthcare professionals working within the emergency medical services system. The methodology and content of the survey were described in a previous publication [20]. The survey was based on an ED safety questionnaire developed in the USA by Magid et al. [21] and modified and validated by the Royal College of Emergency Medicine [22].

The survey is organized into five different safety domains: teamwork, safety leadership, physical environment and equipment, staff and external team, and organizational factors and informatics, with different items in each domain. Seven questions were added to the infection control and team morale domains; these questions were explored in previous studies in different health settings [23]. Each domain is composed of different independent questions, with a total of 36 ordinal scale questions. Each question is based in an orientated assessment where the respondent can select from five different levels of agreement. The study sample included only European countries in which a response rate of more than one valid response per million habitants or, when this criterion was not met, more than 20 responses per country were obtained. A score was elaborated for each domain by the simple addition of the values of questions using the following ranking, ranging from 1 to 5: Never = 1, Rarely = 2, Sometimes = 3, Usually = 4, and Always = 5 (the inverse ranking was used in the negative questions). Higher scores indicated safer situations. The aggregation of the scores for the five safety domains composed the total safety score (TSS).

The country’s health investment per capita (HIPC) and purchasing power parity (PPP) were used to evaluate the effect of the country’s health investment on professionals’ safety perceptions [24]. The response rate was calculated using the most recent available information about country population [25]. The descriptive analysis was performed using central tendency estimators and confidence intervals (95% CI) distribution, the Wald method, and medians [26]. For a clear comparation of the level of safety in each country, the centralized value of the mean (actual value minus mean value) was calculated for the single safety domain. Correlation analysis was used to estimate the associations among the TSS, team morale and infection control scores, and HIPC; the Pearson correlation coefficient was reported. Statistical significance was set at a *p* value of < 0.05. SPSS 17 was used for the analysis. The study did not require research and ethics committee review but received approval from the EUSEM board of directors.

## Results

The initial survey included 1256 responses from 101 different countries, and 1048 responses were finally included in the present study, representing 24 European countries that met the minimum response rate or response rate per million inhabitants. The number of valid responses per country, the rate per population to calculate the TSS, and the HIPC are represented in Table 1.

**Table 1:** Participating countries

Countries	Responses	Rate per population/1 M^a^	Health invest per capita
Albania	17	6.08	350
Austria	28	2.30	6.600
Belgium	46	3.19	5.009
Czech	27	1.33	3.800
Croatia	33	6.90	1.090
Denmark	13	2.20	6.438
Estonia	19	14.26	1.787
Finland	93	10.25	4.600
France	27	0.35	6.110
Germany	43	0.43	5.440
Greece	13	1.217	1.675
Hungary	49	3.61	2.400
Ireland	41	6.63	5.428
Italy	71	0.98	4.030
Malta	20	30.71	2.521
The Netherlands	43	2.07	5.400
Portugal	23	2.21	3.800
Romania	28	1.10	738
Slovenia	16	7.593	2.417
Spain	154	2.68	3.700
Sweden	30	2.37	6.200
Switzerland	46	5.11	9.666
Turkey	31	0.28	1.300
UK	137	1.67	5.380
Total	1048		

^a^Response rate has been calculated using the valid responses, responses that include all the information to calculate the TSS

The descriptive values of the valid responses for each safety domain are shown in Table 2, including the TSS, team morale, and infection control values.

**Table 2:** Safety domains values and other process domains score values

	Survey responses					
	Domain values					
	Theoretical range	*N*	Mean	Min	Max	STD
Safety domains						
Organisational factors and informatics	9–45	913	26.23	11	41	5.37
Physical environment and equipment	5–25	1002	17.08	7	25	3.37
Safety leadership	5–25	947	18.37	7	25	3.63
Staff and external team	6–30	1017	18.23	8	28	3.63
Teamwork	4–20	1021	13.33	5	20	1.97
Total Safety Score (TSS)	36–180	835	93.00	49	137	14.70
Other process domains						
Team morale	5–25	1020	17.42	5	25	3.49
Infection control	2–10	991	7.89	2	10	1.58

The global responses to the individual questions for each domain are summarized in Table 3, including the descriptors of central tendency and variability. Regarding safety, the mean value of responses to some questions was reassuring, such as “enough monitoring devices” (4.20) and “enough personal protective equipment (PPE)” (4.18), while answers to the questions “number of patients” (2.28) and “interruptions affect care” (2.35) scored lowest. The variability among countries in some response is very low, such as “Doctors and nurses work well together” (0.15) or “Handover creates loss of information” (0.22), indicating a diffuse common feeling about these situations. Conversely, items such as “IT resources availability” (0.60) or the questions in the team morale domain show greater variability, with some values exceeding 0.9.

**Table 3:** Survey questions’ responses mean values and variability

Safety questionary			
Domains/questions	Mean	STD	95% CI
Physical environment and equipment			
Responsible is easy to find	3.57	0.26	3.50–3.62
Mental health care	2.98	0.33	2.91–3.05
Enough monitoring devices	4.20	0.39	4.14–4.25
Adequate space	2.90	0.48	2.82–2.97
The ED as safe space	3.43	0.50	3.34–3.46
Safety leadership			
Nonjudgemental environment	3.57	0.37	3.49–3.63
Mentoring young nurses	3.62	0.31	3.55–3.67
Leaders take action	3.64	0.29	3.58–3.71
Protocols on place	3.93	0.40	3.88–4.01
Mentoring young doctors	3.58	0.39	3.53–3.66
Staff and external team			
Sufficient medical staffing	2.60	0.32	2.53–2.65
Timely scans	3.41	0.39	3.35–3.47
Initial assessment works well	3.67	0.29	3.62–3.71
Monitoring vital signs	3.73	0.27	3.66–3.78
Sufficient nurse staffing	2.55	0.40	2.48–2.60
Number of patients	2.28	0.41	2.23–2.34
Organisational factors and informatics			
Pressurised by external targets	2.40	0.36	2.32–2.45
Patients alerts on place	3.45	0.33	3.40–3.52
Hospital procedures support ED flow	2.84	0.27	2.77–2.90
Hospital procedures	2.53	0.26	2.46–259
Hospital information access	3.49	0.42	3.42–3.54
Primary care information access	2.73	0.57	2.64–2.79
Timely transfer to hospital bed	2.48	0.46	2.37–2.50
Friendly use of error report	2.95	0.48	2.87–301
IT resources	3.43	0.60	3.34–3.49
Teamwork			
Interruptions affect care	2.35	0.38	2.29–2.40
Nurses and doctors work well together	3.99	0.15	3.95–402
Handover and loss of information	3.25	0.22	3.19–3.28
Doctors’ communication	3.76	0.22	3.71–3.79
Infection control			
Leaders take action on infection	3.50	0.29	3.46–3.59
Enough PPE	4.18	0.44	4.11–4.22
Team morale			
Less effective	3.24	0.98	3.18–3.30
Proud to work on ED	4.27	0.90	4.22–4.33
Morale in my ED is high	3.24	1.02	3.24–3.17
Good place to work	3.40	0.92	3.35–3.46
Working in my ED is like being part of a large family	3.73	0.99	3.67–3.79

**Fig. 1:**
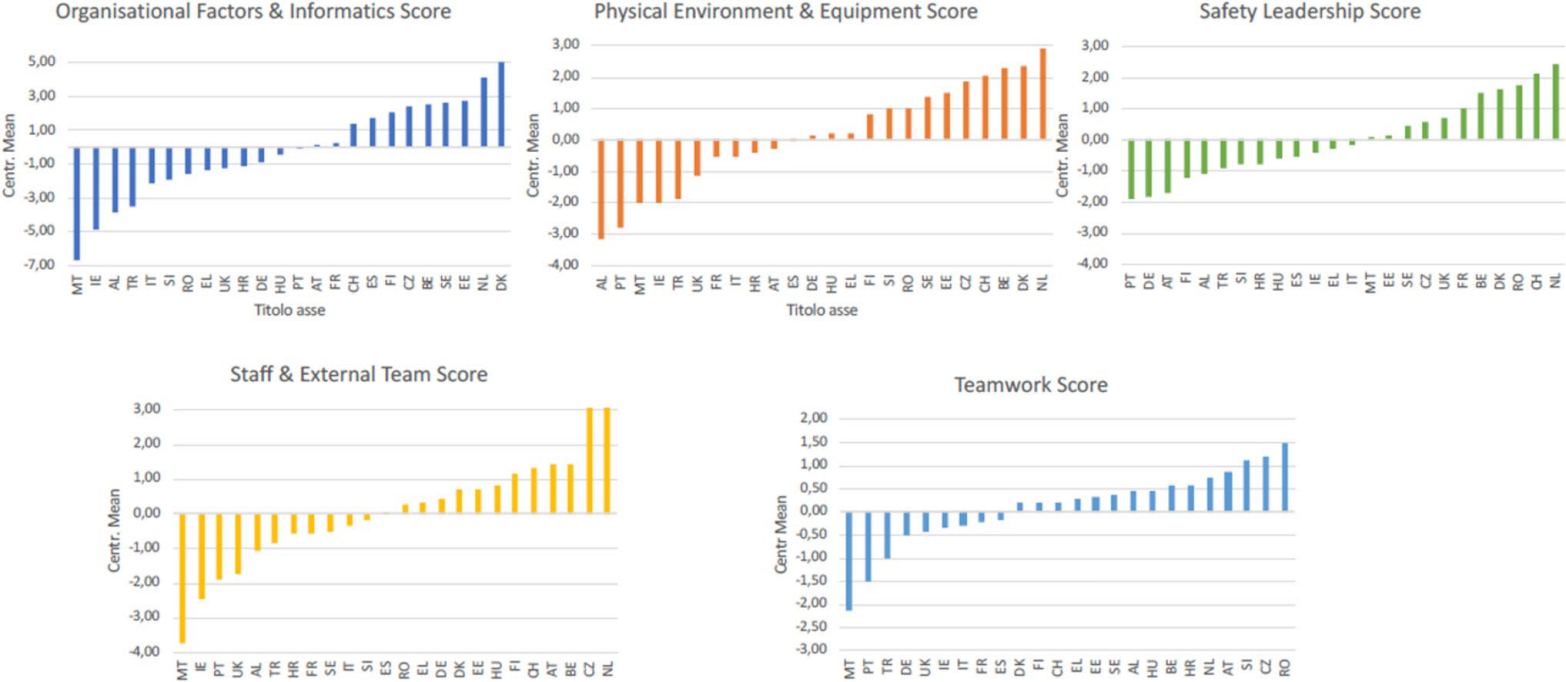
Safety domains’ score per country—*y* axis: total domain score, centralized value of the mean; *x* axis: included countries. *MT* Malta, *IE* Ireland, *PT* Portugal, *UK* United Kingdom, *AL* Albania, *TR* Turkey, *HR* Croatia, *FR* France, *SE* Sweden, *IT* Italy, *SI* Slovenia, *ES* Spain, *RO* Romania, *EL* Greece, *DE* Germany, Denmark DK, Estonia EE, *HU* Hungary, Finland FI, *CH* Switzerland, *AT* Austria, *BE* Belgium, *CZ* check, *NL* Netherland

Differences between countries using the centralized mean values of the different safety domains are shown in Fig. 1.

**Fig. 2:**
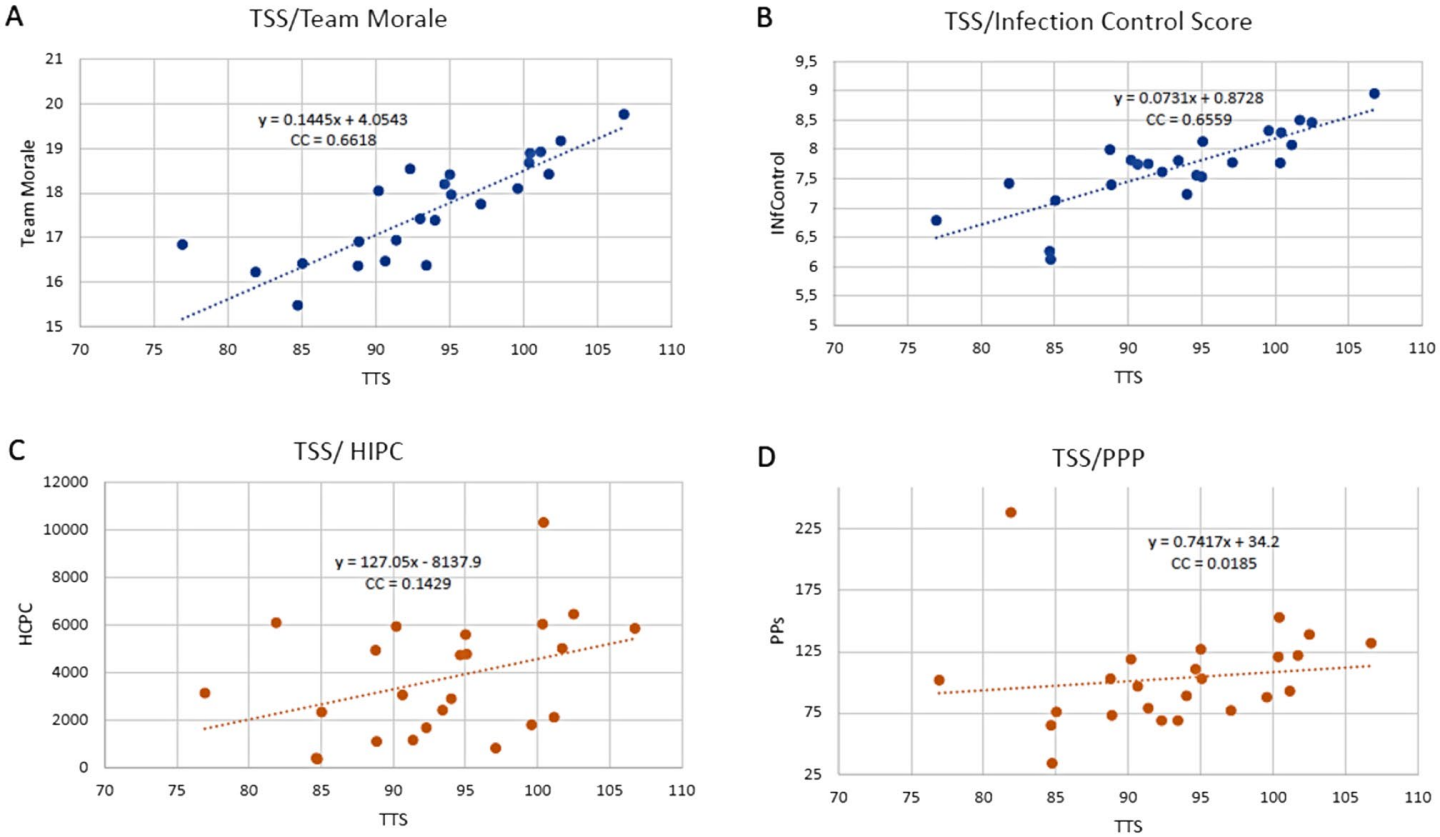
Total Safety Score correlation with: team morale, Infection control, HIPC and PPP. *TSS* total safety score, *HIPC* health invest per capita, *PPP* purchasing power parity

Full details of the responses for each country in each domain, including the team morale and infection control domains, using the mean values are presented in the Appendix. The Pearson correlation coefficient of the TSS for team morale is 0.63 (p < 0.00) and for infection control is 0.62 (p < 0.00). The correlation coefficient of the TTS for HIPC is 0.142 (p > 0.05) and for PPP is 0.136 (p > 0.05) and are represented in Fig. 2.

## Discussion

This study presents a comprehensive assessment of the perception of safety in EDs in 24 European countries. The results highlight the differences between European countries, showing that the TSS of countries is highly variable and indicating that the overall perception of safety is strong in some countries but worrisome in others. However, in each country, there is at least one domain that is more problematic than the others. It is interesting that issues related to safety appear even in wealthy countries with well-functioning healthcare systems.

The countries with higher scores achieved high scores related to the teamwork domain, where the best score was given to “collaboration between nurses and doctors” and “good communication between the team”. Additionally, the availability of monitors and PPE, existence of specific protocols for safe management of patients, and initial assessment and triage were included among the high teamwork scores. These data demonstrate that EDs are well organized and equipped and that the internal organization and competence are strong enough to be considered safe.

The most recurrent safety problems referred to by respondents were overcrowding due to boarding, difficulty in managing patient flow, lack of adequate space, and understaffing (both doctors and nurses). These findings are in line with the European [22, 27–29] and international literature [30–32], although no study has been conducted on European countries.

Management of patients with mental illnesses represents a problem to emergency medicine professionals. This could be due to both insufficient staff and the lack of a suitable space to maintain and manage patients in a safe environment. Moreover, the lack of training on how to deal with such patients may be a source of unsafety. In recent years, there has been considerable interest in mental health and psychiatric illnesses, partly due to the increasing incidence of these illnesses in Europe, particularly in EDs [33–35]. Thus, it is necessary to address this aspect that may exacerbate unsafety and stress among professionals and patients.

The safety domain of organizational factors and informatics, where the questions were related to procedures and support from the hospital and system to ED functioning, seems to be critical and widely heterogeneous among countries. In particular, the procedures for reporting errors are not effective and emergency medicine professionals feel heavy pressure toward external targets, while the hospital may not support ED patient flow and needs. This may be very frustrating and lead to increased burnout and unsafety.

Considering the TSS per country, it is an objective measure of the perception of safety in EDs. Figure 2A, B show the direct correlation between the TSS and the team morale or infection control domain. This means that infection control is a matter of safety concern in EDs [36] and that a safer environment turns into a happier team [37, 38]. A possible suggestion from this observation could be to monitor the TSS over time to measure the impact of safety initiatives and improvements in healthcare systems.

It is noteworthy that safety perceptions in EDs are not closely related to per capita health expenditure, and this is even more evident when the purchasing power of each country is adjusted for, as shown in Fig. 2C, D. This shows that the issue of safety in emergency medicine is quite complex and holds great challenges in Europe, most importantly at the political level. Policymakers and investors are not devoting attention to safety in EDs. On the contrary, there are medium-income countries (e.g., Romania) that have a very high level of safety leadership, physical environment and equipment, and teamwork, demonstrating a marked sensitivity toward emergency medicine safety.

It is evident that each safety aspect in EDs affects the well-being of healthcare workers, reducing burnout and, thus, the rapid turnover of healthcare workers [39]. In addition, safety affects final patient outcomes, reducing the level of mortality and disability and healthcare costs [6].

Healthcare workers and policymakers can use this information to inform strategic planning and decision-making, ultimately leading to improved safety outcomes in emergency medicine in each country and propose European-wide standards.

### Limitations

The study has several limitations, including potential bias in survey responses and variations in healthcare systems and cultural contexts across countries. Future research should explore the reasons behind variations in safety perceptions, examine the impact of safety initiatives on patient outcomes, and identify best practices for improving safety in emergency medicine.

## Conclusions

The data obtained from the safety questionnaire provide valuable insights into the strengths and areas for improvement within emergency medicine in each European country. To improve safety, healthcare institutions should focus on solving the problems identified in each area. This may involve implementing targeted interventions, improving resource allocation, and promoting a safety culture and open communication between healthcare teams. Regular evaluation and monitoring of safety domains can help identify trends and monitor the effectiveness of safety initiatives over time, ultimately leading to a safer and more efficient emergency medicine environment for both patients and healthcare professionals.

### Supplementary Information

Below is the link to the electronic supplementary material.

Supplementary file1 (XLSX 320 KB)

## Data Availability

Data are available in the Appendix.
